# Excluding PTV from lung volume may better predict radiation pneumonitis for intensity modulated radiation therapy in lung cancer patients

**DOI:** 10.1186/s13014-018-1204-x

**Published:** 2019-01-14

**Authors:** Yinnan Meng, Haihua Yang, Wei Wang, Xingni Tang, Caiping Jiang, Yichao Shen, Wei Luo

**Affiliations:** 1 Laboratory of Cellular and Molecular Radiation Oncology, Radiation Oncology Institute of Enze Medical Health Academy, Taizhou, 317000 Zhejiang Province China; 2 Department of Radiation Oncology, Affiliated Taizhou hospital of Wenzhou Medical University, Taizhou, 317000 Zhejiang Province China; 3 Department of Radiation Medicine, University of Kentucky, Lexington, KY 40536 USA

**Keywords:** Lung volume, Radiation pneumonitis (RP), Intensity modulated radiation therapy (IMRT), Lung cancer

## Abstract

**Background:**

Lung dose-volume histogram (DVH) in radiotherapy could be calculated from multiple normal lung definitions. The lung dosimetric parameters generated from various approaches are significantly different. However, limited evidence shows which definition should be used to more accurately predict radiation pneumonitis (RP). We aimed to compare the RP prediction accuracy of dosimetric parameters from three lung volume methods in lung cancer patients treated with Intensity-Modulated Radiation Therapy (IMRT).

**Methods:**

We retrospectively reviewed 183 consecutive lung cancer patients treated with IMRT from January 2014 to October 2017. The normal lungs were defined by total bilateral lung volume (Total Lung), excluding PTV (Lung-PTV) or PGTV (Lung-PGTV). V5, V20, and mean lung dose (MLD) have been extracted from three definitions. The primary endpoint was acute grade 2 or higher RP (RP2). Correlation between RP2 and dose parameters were analyzed by logistic regression. We evaluated prediction performance using area under the receiver operating characteristic curve (AUC) and normal tissue complication probability (NTCP) model.

**Results:**

Twenty-six patients (14.2%) developed acute RP2 after IMRT treatment. Significant dosimetric differences were found between any 2-paired lung volumes (Ps < 0.001). To limit RP2 incidence less than 20%, the cutoff MLDs were 12.5 Gy, 14.2 Gy, and 15.0 Gy, respectively, for Lung-PTV, Lung-PGTV, and Total Lung methods. There were 54% (13% vs. 20%) and 45% (20% vs. 29%) RP2 probability variances detected at each MLD cutoff points from Lung-PTV and Lung-PGTV definitions. The best RP prediction performance was found in MLD from Lung-PTV method (AUC = 0.647), which is significantly better (*P* = 0.006) than the MLD from Lung-PGTV method (AUC = 0.609).

**Conclusion:**

There are significant differences in acute RP2 rate prediction using dosimetric parameters from various normal lung definitions. Excluding PTV from total lung volume may be more accurate and promising to predict acute symptomatic radiation pneumonitis in IMRT treated lung cancer patients.

## Background

Radiation therapy (RT) plays an important role in lung cancer treatment, but grade 2 or higher radiation pneumonitis (RP2) remains an essential dose-limiting obstacle and can significantly reduce the therapeutic ratio and patient’s quality of life [1–3]. Lung volume receiving more than 20 Gy (V20) and mean lung dose (MLD) generated from dose-volume histograms (DVHs) are the most common traditional dosimetric parameters in clinical treatment planning evaluation [4–6]. Some retrospective analyses have also correlated radiation pneumonitis with low-dose parameters, such as the V5 [7, 8].

For the past decade, three-dimensional conformal external beam radiation therapy (3D-CRT) with concurrent chemotherapy was commonly to treat unresectable local advanced lung cancer, but there has been increasing use of intensity-modulated radiation therapy (IMRT) [9–11]. In the 2018 NCCN guideline in NSCLC version 4 updates, according to a secondary analysis of RTOG 0617, the IMRT is currently recommended as a preferred RT technique over 3DCRT [12]. IMRT group was associated with a decrease of grade ≥ 3 RP incidence from 7.9 to 3.5% with similar survivals and tumor control outcomes, despite larger tumors, higher V5 s, similar V20 s, and MLDs [13]. The available dose constraints are generally from previous 3D-CRT studies, due to the more unconstrained beam arrangements and nonstandard dose distribution, they may have lower radiation pneumonitis prediction value for the IMRT [5, 6]. More precise dose constraints for the IMRT technique need to be further developed instead of using the conventional constraints from 3D-CRT.

Moreover, the normal lung volume definitions for DVH calculation are found inconsistent in the previous studies, which could have a significant impact on the variance of dose parameters and the evaluation of clinical treatment decision [14, 15]. In RTOG 0617 the lung volume was defined as the bilateral lung volume excluding the CTV (Lung-CTV). It was more commonly defined as the bilateral lung excluding the planning target volume (Lung-PTV) [5, 16, 17] or excluding the gross tumor volume (Lung-GTV) [1, 3, 4]. Currently, both RTOG and ESTRO-ACROP guidelines recommend using Lung-GTV delineation instead of Lung-PTV to standardize lung volume definition among different institutions [18, 19]. But limited clinical evidence shows which normal lung definition is better for symptomatic radiation pneumonitis prediction, especially using IMRT or VMAT which has a non-standard dose distribution outside treatment target.

In the IMRT era for lung cancer patients, we hypothesized that a significant dosimetric parameters variation could still be found among different total lung definitions, and a specific normal lung defined method could be superior to the others in RP2 prediction. In our study, we compared the numeric dose difference and the acute RP2 prediction performance among dose parameters from three normal lung definitions.

## Methods

### Patients

This study was approved by the Institutional Review Board, which waived written informed consent because of the retrospective design. We retrospectively reviewed 183 lung cancer patients received IMRT at our institution between January 2014 and September 2017. The inclusion criteria were the first time receiving thorax RT, having received only IMRT technique with RT alone or combine with either surgery or chemotherapy, prescription dose PGTV≥50 Gy in 2.00–2.20 Gy and PTV ≥ 45 Gy in 1.80 Gy daily fractions using 6 MV photons, having available dosimetric data, and having follow-up records for at least 3 months.

Image-guided RT including orthogonal megavoltage electronic portal imaging or kilovoltage cone beam computed tomography (CBCT) was used to reduce the interfraction geometric displacement from the daily setup error and the anatomic change. The patients with a mid-treatment computed tomography (CT) scan and a replanning adaptive radiotherapy were not included in this study. Dosimetric factors evaluated were V5, V20, and MLD from three normal lung definitions. Clinical factors analyzed including age, gender, smoking status, tumor histology and stage, receipt of chemotherapy or surgery, target prescription and volume.

### Treatment planning

Treatment planning CT scans were performed with patients in the treatment position, immobilized in the supine position with their arms above their head. Scans should include the entire thorax for at least 5 mm slice thickness. The pretreatment positron emission tomography (PET)/CT may be used in staging and tumor volume delineation. The 4D CT or 4D PET/CT scan has not been applied to the patients receiving IMRT treatment in our department.

Gross tumor volume (GTV), defined as visible primary tumor and positive metastatic lymphadenopathy treatment planning CT or pretreatment PET scan. Clinical target volume (CTV) was defined as GTV with a 0.5 cm to 1 cm margin combined with positive lymph node involved region to cover the microscopic tumor extension. The GTV margin was approximate 6 mm for squamous cell or 8 mm for other types. The tumor and target volumes were contoured by treating physicians under the supervision of a senior radiation oncologist. The planning gross tumor volume (PGTV) and planning target volume (PTV) was defined as the GTV and CTV with a 5 mm uniform expansion to account for the setup margin.

RT planning was done using Pinnacle treatment planning system (Philips Medical Systems, Andover, MA) with Collapsed Cone Convolution algorithm or Eclipse software (Varian medical systems, Palo Alto, CA) with Analytical Anisotropic Algorithm. The radiation dose distribution was calculated using lung heterogeneity corrections.

### Lung volume definition and lung DVH

Lung was contoured in CT datasets using pulmonary windows via threshold auto-segmentation followed by manual edits. All inflated, collapsed, fibrotic, and emphysematous lung tissues were contoured with the inclusion of small vessels in the lung parenchyma. Great vessels, trachea, and proximal bronchial tree were excluded.

**Fig. 1:**
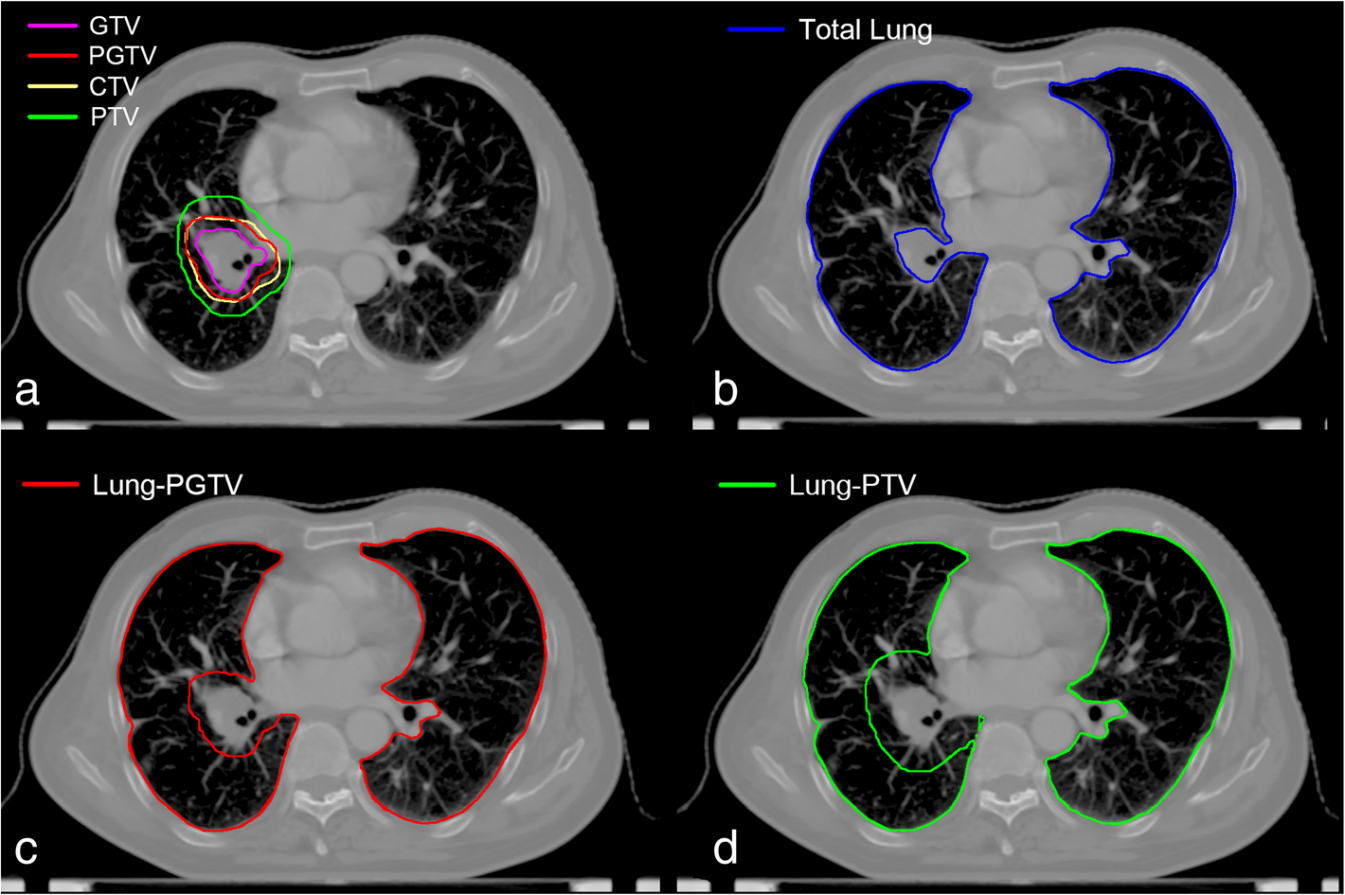
Contouring examples of targets and three normal lung definitions. **a** GTV = Gross Tumor Volume; PGTV = Planning Gross Tumor Volume; CTV = Clinical Target Volume; PTV = Planning Target Volume. **b** Directly using total bilateral lung volume definition. **c** Excluding PGTV from total bilateral lung volume definition. **d** Excluding PTV from total bilateral lung volume definition

Three sets of normal lung DVHs were generated by using the total bilateral lung volume (Total Lung), with the exclusion of targets of planning GTV from bilateral lung (Lung-PGTV) and PTV (Lung-PTV) (Fig. 1). Target exclusion was performed by overlapping rules (i.e., only the intrapulmonary parts of targets were subtracted). From each bilateral lung DVH, three dosimetric factors were extracted: V5, V20, and MLD. The V5/20 was defined as the percentage of total normal lung volume receiving equal to or greater than 5/20 Gy of radiation.

### Evaluation of radiation pneumonitis

RP was diagnosed and graded based on clinical and radiographic presentations, according to the National Cancer Institute’s Common Terminology Criteria for Adverse Events, version 4.03 [20]. In brief, diagnosis of RP required the presence of radiographic pneumonitis not attributable to other causes such as infection or tumor recurrence.

Grade 1 pneumonitis was radiographic RP with no or minimal symptoms that did not require medical intervention; grade 2 was symptomatic but did not interfere with daily activities; grade 3 was symptomatic and interfered with daily activities or required administration of oxygen to the patient; grade 4 required assisted ventilation for the patient; and grade 5 pneumonitis was fatal. A symptomatic RP event for analysis was defined as RP2. The endpoint for this analysis was acute RP2 happened ≤3 months.

### Lyman NTCP model parameters

We built the MLD-RP2 association curve using the Lyman model. The dosimetric and RP2 data from 183 patients were used to fit the NTCP model. When volume-effect parameter *n* = 1, the NTCP equation reduces to an expression representing the mean lung dose. We use the log-likelihood function to get best-fit parameters m and TD50 [21].

### Statistical analysis

For description, we used the mean and 95% confidential interval (CI) for normal distribution continuous variables, median and range for non-normal continuous variables; categorical variables are reported as count and percentage. Univariate logistic regression analysis was performed to evaluate the correlation between clinical and dosimetric factors with the onset of acute symptomatic RP. Dosimetric factors from the different methods DVH calculation were compared using repeated analysis of variance test (ANOVA). Dose difference evaluation is presented as mean and 95% CI. The difference of dosimetric factors between acute RP2 and non-RP2 patients were compared with the Mann-Whitney U test (Equivalent with Wilcoxon rank-sum test). The area under the curve (AUC) of receiver operating characteristic (ROC) curves were calculated to quantify the ability of the various V5, V20, and MLD. The increase in the AUC was evaluated for significance using the test proposed by DeLong et al. [22]. SPSS, version 23.0 (IBM, Armonk, NY) and MedCalc, version 14.8.1 (MedCalc Software bvba, Ostend, Belgium) were used. Differences were considered significant if *P* < 0.05 (2- sided).

## Results

### Patient’s characteristics

From January 2014 to October 2017, 183 consecutive patients with follow-up lung toxicity data were evaluated. Patients were treated with curative or palliative intent with RT alone or combine with either surgery or chemotherapy. The prescription dose was between 50 and 70 Gy. All of these patients were only treated by IMRT technique. Twenty six patients (14.2%) developed acute RP2 within 3 months after treatment. No patient suffered from grade 5 RP. Table 1 shows the clinical characteristics and their correlation with the development of RP2. None of the baseline clinical factors, including age, gender, smoking history, clinical stage, PGTV or PTV volume, prescription dose, chemo, surgery, BMI or target volume has statistical significance with the risk of acute RP2.

**Table 1:** Baseline and clinical characteristics of patients and their correlation with grade ≥ 2 acute radiation pneumonitis

Characteristics	No. of Patients (*N* = 183) (%)	No. of Grade ≥ 2 RP (*N* = 26) (%)	Odds Ratio	95% CI	*P* Value*
Age					
≤ 63 (Median)	98 (53.6)	13(50.0)	Reference		
> 63 (Median)	84(45.9)	13(50.0)	1.21	0.53–2.78	0.651
Gender					
Male	162(88.5)	20(76.9)	Reference		
Female	21(11.5)	6(23.1)	2.84	0.99–8.17	0.053
Smoking					
Never	42(23.0)	7(26.9)	Reference		0.644
Current	77(42.1)	12(46.2)	0.92	0.33–2.56	0.878
Former	64(35.0)	7 (26.9)	0.61	0.20–1.90	0.397
Pathology					
Squamous	112(61.7)	19 (73.1)	Reference		0.570
Adenocarcinoma	30(16.4)	3(11.5)	0.54	0.15–1.98	0.355
Small Cell	35(19.7)	3(11.5)	0.46	0.13–1.65	0.234
Others	6(2.2)	1(3.8)	0.98	0.11–8.86	0.985
Stage					
I/II	14(7.7)	0(0)	0.00		0.999
III	115(62.8)	20 (76.9)	Reference		0.578
IV	54(29.5)	6 (23.1)	0.59	0.22–1.58	0.295
Chemo					
No	25(13.7)	1(3.8)	Reference		
Yes	158(86.3)	25(96.2)	4.51	0.58–34.89	0.149
Surgery					
No	139(76)	20(76.9)	Reference		
Yes	44(24)	6(23.1)	0.94	0.35–2.51	0.901
PGTV Volume					
≤ 135.1 (Median)	92(50)	13(50)	Reference		
> 135.1 (Median)	91(50)	13(50)	1.0	0.44–2.32	0.976
PTV Volume					
≤ 543.6 (Median)	92(50)	17(65)	Reference		
> 543.6 (Median)	91(50)	9(35)	0.48	0.20–1.15	0.101
PGTV prescription					
≤ 54 (Median)	113(62)	14(54)	Reference		
> 54 (Median)	70(38)	12(46)	1.46	0.63–3.38	0.373
PTV prescription					
≤ 50 (Median)	161(88)	23(88)	Reference		
> 50 (Median)	22(12)	3(12)	0.95	0.26–3.46	0.935

*Abbreviation: PTV* planning target volume, *PGTV* planning gross tumor volume, *V5/20* volume of lung receiving a dose ≥5/20 Gy, *MLD* mean lung dose

*By repeated analysis of variance test (ANOVA)

### Difference of lung dose from three lung volume definitions

The mean dosimetric value comparison of V5, V20 and MLD among the three lung volume contouring methods are shown in Table 2. Repeated measures ANOVA shows a significant difference between any 2-paired methods (Ps = 0.000). The mean value difference for Lung-PTV definition from the other two methods is relatively large. The difference between Lung-PGTV and total lung methods is small but significant.

**Table 2:** Difference of dosimetric factors among three lung definition methods

Factors		Mean Difference	95% CI	*P* Value*
V5 (%)	Lung-PTV vs. Lung-PGTV	2.2	2.0–2.4	< 0.001
	Lung-PTV vs. Total Lung	2.7	2.5–3.0	< 0.001
	Lung-PGTV vs. Total Lung	0.6	0.4–0.7	< 0.001
V20 (%)	Lung-PTV vs. Lung-PGTV	3.5	3.3–3.8	< 0.001
	Lung-PTV vs. Total Lung	4.5	4.2–4.8	< 0.001
	Lung-PGTV vs. Total Lung	1.0	0.8–1.1	< 0.001
MLD (Gy)	Lung-PTV vs. Lung-PGTV	1.7	1.6–1.8	< 0.001
	Lung-PTV vs. Total Lung	2.3	2.1–2.4	< 0.001
	Lung-PGTV vs. Total Lung	0.6	0.5–0.7	< 0.001

*Abbreviation: PTV* planning target volume, *PGTV* planning gross tumor volume, *V5/20* volume of lung receiving a dose ≥5/20 Gy, *MLD* mean lung dose

*By repeated analysis of variance test (ANOVA)

### Correlation of dosimetric factors with RP2

The difference of dose between acute RP2 patients and non-RP2 patients are shown in Table 3. From the Mann-Whitney U test, all dosimetric parameters from Lung-PTV method have a significant difference between RP2 and non-RP2 patients (Ps < 0.05). For Lung-PGTV method, only the V20 difference is significant (*P* = 0.046). None of the parameters from Total Lung method shows a significant difference between the two groups.

**Table 3:** Difference of dosimetric factors between RP2 and non-RP2 groups

Factors	Non-RP2		RP2		*P* Value*
	Median	Range	Median	Range	
Lung-PTV method					
V5 Lung-PTV (%)	48	23–76	50.5	31–78	0.046
V20 Lung-PTV (%)	18	8–26	19	15–31	0.035
MLD Lung-PTV (Gy)	10.3	6.0–15.1	11.3	7.9–15.6	0.016
Lung-PGTV method					
V5 Lung-PGTV (%)	51	26–76	52.5	33–80	0.085
V20 Lung-PGTV (%)	22	11–31	24	18–34	0.046
MLD Lung-PGTV (Gy)	12.1	7.0–17.0	13.1	9.7–18.1	0.076
Total Lung Method					
V5 Total Lung (%)	52	27–77	53	36–80	0.091
V20 Total Lung (%)	23	11–31	24	20–36	0.080
MLD Total Lung (Gy)	12.6	7.2–17.3	13.7	10.1–19.6	0.097

*Abbreviation: RP2* radiation pneumonitis≥ grade 2, *PTV* planning target volume, *PGTV* planning gross tumor volume, *V5/20* volume of lung receiving a dose ≥5/20 Gy, *MLD* mean lung dose

*By Mann-Whitney U test

From logistic regression analysis, all of the dosimetric factors from three methods are correlated with the incidence of RP2 (all Ps < 0.05). But each parameter from Lung-PTV method shows a stronger correlation evidence (smaller Ps) compared with the parameters from the other two lung volume definitions (Table 4).

**Table 4:** Univariate analysis of dosimetric factors related to the occurrence of grade ≥ 2 acute radiation pneumonitis

Factors	Odds Ratio	95% CI	*P* Value*
V5 Lung-PTV	1.054	1.014–1.096	0.008
V5 Lung-PGTV	1.048	1.007–1.091	0.023
V5 Total Lung	1.049	1.007–1.094	0.022
V20 Lung-PTV	1.204	1.059–1.369	0.005
V20 Lung-PGTV	1.164	1.039–1.305	0.009
V20 Total Lung	1.137	1.015–1.272	0.026
MLD Lung-PTV	1.421	1.116–1.809	0.004
MLD Lung-PGTV	1.271	1.038–1.557	0.021
MLD Total Lung	1.232	1.012–1.501	0.038

*Abbreviation: CI* confidence interval, *PTV* planning target volume, *PGTV* planning gross tumor volume, *V5/20* volume of lung receiving a dose ≥5/20 Gy, *MLD* mean lung dose

*By univariate logistic regression analysis

### RP2 prediction evaluation

**Fig. 2:**
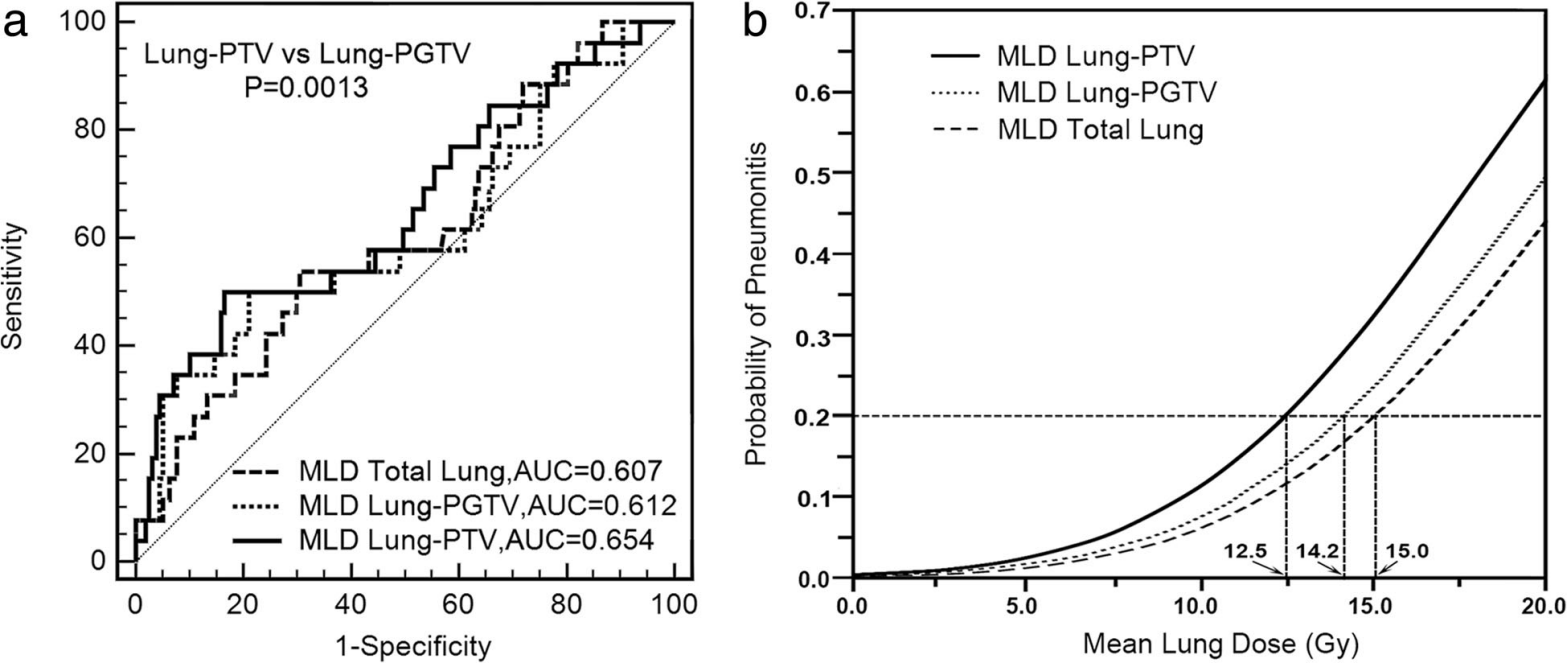
**a** Receiver operating curves for mean lung dose (MLD) associated with the grade ≥ 2 radiation pneumonitis from three normal lung definitions. **b** The mean lung dose and the grade ≥ 2 radiation pneumonitis relationship models from three normal lung definitions

We used ROC curve analysis to evaluate dose parameters RP2 prediction ability. MLD from Lung-PTV method has the highest AUC value of 0.647. Total lung method parameters have the smallest AUC value among the three methods. All AUC values of factors from Lung-PTV are higher than Lung-PGTV method. Comparing with Lung-PGTV, according to Delong test [22], Lung-PTV method has a significant AUC increase in V5 (*P* = 0.001) and MLD (*P* = 0.006). To limit RP incidence less than 20% according to these NTCP models, the MLD cutoff points are 12.5 Gy, 14.2 Gy, and 15.0 Gy for Lung-PTV, Lung-PGTV, and Total Lung, respectively. Comparing Lung-PTV and Lung-PGTV method, the incidence probability has 54% difference (13% vs. 20%) at MLD = 12.5 Gy, and 45% (20% vs. 29%) difference at MLD = 14.2 Gy (Fig. 2).

## Discussion

The current study found that different normal lung definitions could have a significant impact on V5, V20 and MLD for IMRT treated lung cancer patients. This finding is in line with the previous results from 3D-CRT [14, 15, 23], indicating that the choice of lung definitions should not be disregard in clinical IMRT practice. According to NTCP models, Lung-PTV and Lung-PGTV methods could have a 1.7 Gy MLD difference and up to 54% probability disagreement when limiting RP2 incidence under 20%. We also found that dosimetric parameters, not clinical variables, are significantly correlated with RP2 development. All V5, V20, and MLD from Lung-PTV definition show stronger evidence in the correlation with RP2 development than the other two definitions. It is interesting to note that V5 and MLD from Lung-PTV definition could significantly improve the RP2 prediction performance compared with those from Lung-PGTV method based on our ROC curves analysis.

To our knowledge, this is the first study to demonstrate the impact of different lung volume definitions on dosimetric parameters on IMRT treated lung cancer patients. We further analyze which lung defined method has a better performance in RP2 correlation and prediction. The magnitudes of the dose differences from our results could have a significant clinical impact. In the Quantitative Analysis of Normal Tissue Effects in Clinic (QUANTEC) lung project, over 70 published articles were reviewed but dosimetric data were calculated from inconsistent lung volume definitions [6]. The dose-RP2 relationship disparity from various studies could be reduced by overcoming the incompatible lung volume define methods. A meta-analysis by Palma et al. used a linear imputation equation to the convert V20 and MLD between Lung-PTV and Lung-PGTV [24]. In this way, they could convert data from either a method or another. However, from our experience, the PTV is exceedingly different from a patient to another. A simple fit equation could not accurately convert dosimetric parameters between two methods for a specific patient, especially with a PTV size far from the mean value.

Wang et al. showed that the lung-PGTV method seems to provide more accurate lung toxicity prediction [14] than the Lung-PTV method. Several reasons may cause this opposite finding to our result: First, the CTV defined in their RP2 prediction results was contoured with a 0.8 cm uniform expansion directly from GTV instead of the CTV including involved lymph node regions contoured by a physician in our study. Excluding an expanded GTV and excluding clinical prescribed PTV are two inherently distinguished methods, which will provide different calculated DVHs. Second, we included stage IV palliative radiation therapy patients in our study, the treatment prescription has a broader dose spectrum. Third, we included the postoperative patients, which don’t have a PGTV. The only treatment targets in these cases are the PTVs. Furthermore, all of our treatment were using IMRT instead of 3DCRT, the nonstandard dose distribution might contribute to this opposite result.

Treatment plans usually have a goal of delivering a prescribed dose to at least 95% of the PTV for both 3D-CRT and IMRT techniques. Admittedly, the dose heterogeneity index within the PTV could be diverse for these two techniques [25], the PTV dose coverage is always around 95%. The main dose distribution difference between these two techniques is located in the region outside the treatment target volume, as IMRT tends to deliver a higher conformable dose at the expense of irradiating larger lung volume. A specific lung cancer patient plan using IMRT could have similar V20 and MLD compared with using 3D-CRT while having two entirely different shapes of DVHs. These different histogram shapes primarily come from the dose distribution disparity outside PTV, and it is very likely contributing to various lung toxicity outcome.

We recognized that this study is limited in several aspects. First, treatment plans were calculated in two different treatment planning systems. The dose-volume histogram parameters could be marginally different if a patient’s dose was calculated using another algorithm. Second, it was a single institution retrospective study, 183 IMRT treated patients were relatively a small sample size, considering there were only 26 acute RP2 patients. Nevertheless, this study validated the non-negligible impact of different lung definitions on dose and RP2 prediction using IMRT technique. Technique similarity and normal lung definitions should be taken into consideration before using specific dose constraints in clinical practice and decision making. We identified dosimetric parameters from Lung-PTV method provide stronger evidence in the correlation of RP2, and better RP2 prediction performance.

## Conclusions

The same dose constraint from three normal lung definitions could predict significant different RP2 rate. The Lung-PTV method may be better than or at least as good as Lung-PGTV method regarding RP2 prediction. We recommend adding dose constraints from Lung-PTV method in clinical treatment plan evaluation. Since the dose distribution differences among multiple techniques mainly locate in the lung region outside PTV, dosimetric data from Lung-PTV method could help us further study the more precise lung dose constraints for currently common techniques like IMRT.

## Abbreviations

3D-CRT: Three-dimensional Conformal Radiation Therapy
ANOVA: The Analysis of Variance
AUC: Area Under the Receiver Operating Characteristic Curve
BMI: Body Mass Index
CI: Confidential Interval
CTCAE: Common Terminology Criteria for Adverse Events
CTV: Clinical Target Volume
DVH: Dose-volume Histogram
IMRT: Intensity Modulated Radiation Therapy (IMRT)
Lung-PGTV: Excluding PGTV from Bilateral Lung
Lung-PTV: Excluding PTV from Bilateral Lung
MLD: Mean Lung Dose
NTCP: Normal Tissue Complication Probability
PTV: Planning Target Volume
ROC: Receiver Operating Characteristic
RP: Radiation Pneumonitis
RP2: Grade 2 or Higher RP
TD50: Toxicity Occurs in 50% of the Population
V5/20: The Percentage of Lung Volume Receiving Equal to or Greater than 5 or 20 Gy
VMAT: Volumetric Modulated Arc Therapy
